# The landscape of ketamine use disorder: Patient experiences and perspectives on current treatment options

**DOI:** 10.1111/add.70073

**Published:** 2025-04-28

**Authors:** Rebecca E. Harding, Tamsin Barton, Maeve Niepceron, Ella Harris, Emily Bennett, Emily Gent, Flora Fraser, Celia J. A. Morgan

**Affiliations:** ^1^ Clinical Psychopharmacology Unit University College London Gower Street London UK; ^2^ Psychopharmacology and Addiction Research Centre, University of Exeter Exeter UK

**Keywords:** addiction, dependence, ketamine, non‐treatment‐seeking abstinence, survey, treatment, treatment‐seeking

## Abstract

**Aims:**

To report the symptoms and aetiology of ketamine use disorder (KUD), gauge the effectiveness of current treatment services and identify strategies to enhance patient access and outcomes.

**Design:**

Mixed‐methods, cross‐sectional questionnaire. Electronic survey from November 2023 to April 2024.

**Setting:**

Participants were recruited through snowball sampling, social media and referrals from UK addiction treatment services. The survey was open to international participants, with responses collected from the United Kingdom, United States, Canada, Europe and Australia.

**Participants/Cases:**

A total of 274 individuals with self‐identified KUD, including both treatment‐seeking (40%) and non‐treatment‐seeking (60%) current or former ketamine users. Participants' ages ranged from 18 to 67 years old, with 47.7% identifying as male. Additionally, 58.8% reported a diagnosed mental health disorder. On average, participants consumed 2.0 g of ketamine per day, with treatment‐seeking individuals reporting higher average use (M = 2.67 g) than non‐treatment‐seeking users (M = 1.68 g) (*P* < 0.001).

**Measurements:**

Participants completed an online questionnaire addressing their attitudes toward ketamine and treatment services, including questions pertaining to their symptoms of problematic ketamine use, perceptions of education and awareness about KUD, opinions of existing treatment options, and facilitators for seeking treatment.

**Findings:**

The study identified various physical symptoms associated with KUD, with bladder problems (60%), nasal problems (60%) and ‘K‐cramps’ (56%) being commonly reported among all users. In response to these symptoms, the majority (56%) did not seek treatment; among treatment‐seeking users only 36% reported feeling satisfied with their care. Symptoms of abstinence syndrome were also identified, including cravings (71%), low mood (62%), anxiety (59%) and irritability (45%). Treatment‐seeking participants reported that the services they used had little (31%) or some (31%) awareness of ketamine, were not tailored to ketamine use (43%) and were generally only somewhat effective (43%). Fifty‐nine percent of participants reported that there was “definitely not” sufficient awareness in education and peer groups about the risks associated with ketamine use. When asked about the most important factors when choosing a treatment program, cost/affordability was the most cited for all participants.

**Conclusions:**

Ketamine use disorder (KUD) appears to be associated with a high prevalence of physical and psychological symptoms, including some specifically linked to abstinence. Despite this, most individuals with KUD do not seek treatment, and existing services are often perceived as ineffective.

## INTRODUCTION

Ketamine, a glutamatergic *N*‐methyl‐d‐aspartate receptor antagonist, has well‐established clinical value, particularly in anaesthesia and pain management [1]. More recently, the transdiagnostic potential of ketamine in psychiatry has garnered significant attention [2, 3]. Typically, ketamine is administered in a tightly controlled clinical setting, often accompanied by a psychotherapy protocol. However, the growing trend of telemedicine‐facilitated at‐home ketamine therapy, particularly in the United States (US), has heightened concerns about its abuse potential. Recognising this, the US Food and Drug Administration has issued a compounding risk alert, acknowledging the potential for abuse [4]. Alarmingly, recent data indicate that more than 50% of individuals prescribed ketamine have exceeded the recommended dosage, either accidentally or intentionally [5]. These developments underscore the critical need to understand the risks associated with ketamine, particularly its potential for abuse and addiction.

Beyond clinical use, recreational ketamine consumption has surged. Perhaps owing to its short‐acting, euphoric and hallucinogenic effects, ketamine has become the fourth most commonly used drug in clubbing environments in the United Kingdom (UK) [6, 7]. Likewise, US data show a 1116% increase in the total weight of ketamine seized between 2017 and 2022, suggesting a sharp rise in non‐medical or recreational use [8]. Government data from the UK indicate that ketamine use has more than doubled since 2016, with a threefold increase among individuals under 25 years old [7]. Concurrently, the number of adults requiring treatment for ketamine use disorder (KUD) has risen fivefold since 2015 [9]. Despite the growing prevalence and associated burden on healthcare services, significant gaps in knowledge remain concerning KUD, particularly regarding the extent of its addictive potential and effective treatment strategies.

Research has increasingly highlighted the severe psychological and physical consequences of chronic ketamine use. Studies have linked it to widespread structural and functional changes in the brain, including global reductions in grey matter volume [10]. Likewise, KUD has been linked to significant cognitive impairments [11] and individuals with KUD frequently report increased depressive symptoms [12]. One of the most common physical complications of KUD is ketamine‐induced cystitis, with symptom severity correlating positively with ketamine dosage [13]. Aside from its urological effects, ketamine use is also associated with gastrointestinal issues, with previous studies reporting that approximately 25% of users experience symptoms such as abdominal pain (‘K‐cramps’) [14]. These discomforts often lead to increased ketamine consumption in an attempt to alleviate pain [1]. Although these significant physical and psychological consequences exist, only 50% of those affected seek treatment for their bladder issues [13], and even fewer pursue treatment for KUD itself, suggesting significant barriers to accessing effective care.

Currently, there is a critical gap in research exploring the perceptions of individuals with KUD regarding their symptoms and treatment experiences. To address this gap, we conducted an exploratory, mixed‐methods, cross‐sectional online questionnaire targeting current and former problematic ketamine users. Our research aims to inform treatment programs, improve public understanding and facilitate better access to effective care for those affected by KUD.

## METHODS

### Participants and recruitment

Participants were eligible if they were fluent in English (although international responses were welcome) and had a current or previous history of problematic ketamine use. Initially, 375 participants consented, however, 86 were excluded because of not reporting KUD. Participants were required to be over 18 as the treatment needs and healthcare access for minors are distinct, often involving parental consent and specialised services, making it difficult to generalise findings to an adult population. Therefore, four adolescent participants were excluded. The final sample consisted of 274 individuals, with 179 completing all survey items.

Rather than conducting an *a priori* power analysis in this exploratory study, we aimed to maximise data collection, given the unknown prevalence of KUD and recruitment challenges. While we did not specify a predetermined target, the final sample size provides sufficient variability for this present analysis.

The online survey, administered via Qualtrics, was available between November 2023 and April 2024. Participants were recruited via snowball sampling, social media platforms and specific Reddit threads focused on ketamine addiction (*n* = 209). A smaller subset was recruited through UK addiction treatment services, including Humankind and Change Grow Live. No financial compensation was provided.

### Procedure

The study was approved by the Local Research Ethics Committee at the University of Exeter. Informed consent was obtained from all participants. Using a cross‐sectional survey design, participants were assigned to separate question streams based on treatment‐seeking status: 52 questions for those seeking treatment and 48 for those who were not. This was an original questionnaire, developed with expert input from treatment service providers and piloted with five individuals with KUD, which used a mixed‐methods approach to gather qualitative and quantitative data.

### Measures

For the full list of questions, see Data S1. The survey collected demographic data, and classified participants as treatment‐seeking or non‐treatment‐seeking (either current or prior) for KUD. Additional data were collected on ketamine usage patterns, including frequency (days per week), quantity consumed, method of consumption and the recency of last use.

Multiple‐choice, Likert‐scale questions and open‐ended and closed‐ended questions explored motivations for ketamine use, symptoms of problematic use and abstinence symptoms. Treatment‐seeking participants provided details on treatments tried, frequency of attempts and perceived effectiveness (rated 0–4, from ‘not at all’ to ‘very effective’). Additional questions for this group focused on the primary reasons for seeking addiction treatment and perceptions of KUD awareness among treatment providers. All participants rated the perceived effectiveness, accessibility, affordability of treatment, as well as stigma, awareness of KUD, and trust in addiction treatment services, using a 5‐point Likert scale (0–5, from ‘strongly disagree’ to ‘strongly agree’). Open‐ended questions explored barriers to treatment, factors that facilitated treatment access and desired improvements in treatment services for both treatment‐seeking and non‐treatment seeking participants.

### Analysis

Given participant attrition, the number of respondents per question was reported, ensuring maximum data retention. It was not appropriate to impute missing data given the nature of the data collected. Descriptive statistics were used to summarise the study sample. Thematic analysis of qualitative data followed structured tabular thematic analysis (ST‐TA) [15], coding common language used by participants to identify themes and assess their frequency across open‐ended questions. Group differences in demographic measures and characteristics of ketamine use between treatment‐seeking and non‐treatment‐seeking individuals were examined using χ^2^ tests for categorical variables and independent *t* tests for continuous variables. Welch's *t* test was applied when Levene's test indicated unequal variances between groups, with adjusted degrees of freedom reported accordingly. Significance was set at *P* < 0.05. Quantitative analyses were conducted using SPSS.

## RESULTS

### Demographics

The majority of the sample (*n* = 274) consisted of current ketamine users (68.7%). A substantial proportion of participants identified as non‐treatment‐seeking (*n* = 131, 59.82%) (Table 1). The gender distribution was 47.7% male, 44.2% female, 4.3% transgender male and 3.9% identifying as other. Most participants (75%) were from the UK, and 58.8% reported having a diagnosed mental health disorder. The survey was predominantly accessed online (74%), with smaller percentages finding it through treatment services (3%) or via family and friends (18%). The average age of participants was 28 years in both treatment‐seeking and non‐treatment‐seeking groups, with an age range of 18 to 67 years. No demographic differences emerged between treatment and non‐treatment seeking groups (see Table 1 for analyses). Notably, there was a significantly larger amount used per day in the treatment‐seeking group and non‐treatment seeking group (t_152_ = 3.73, *P* < 0.001).

**TABLE 1:** Demographics and characteristics of ketamine use among treatment‐seeking and non‐treatment‐seeking participants.

Characteristics	Treatment‐seeking (*n* = 87)		Non‐treatment‐seeking (*n* = 127)		Statistical test
Gender (female %)	39 (45)		57 (45)		χ^2^ (1) = 0.00, *P* = 1.00
Mental health diagnosis (%)	55 (63)		74 (58.3)		χ^2^ (1) = 0.17, *P* = 0.678
Location, UK (%)	65 (75)		88 (69)		χ^2^ (1) = 0.69, *P* = 0.407
	**Mean (SD)**	**Range**	**Mean (SD)**	**Range**	
Age (y)	27.39 (8.52)	18–59	27.90 (7.93)	18–67	t_212_ = −0.44, *P* = 0.659
How old were you when you first tried ketamine? (y)	18.95 (6.02)	12–50	19.60 (5.26)	15–45	t_212_ = −0.82, *P* = 0.416
How many days ago did you last take ketamine?	178.12 (1098.77)	0–9618	50.06 (213.24)	0–1800	t_115_ = 1.07, *P* = 0.286
How frequently do you use ketamine? (out of 7 days a week)	5.44 (1.76)	0–7	5 (1.92)	1–7	t_212_ = 1.73, *P* = 0.085
How much ketamine do you take a day when you use? (g)	2.67 (1.80)	0.25–10	1.68 (2.05)	0.01–20	t_152_ = 3.73, *P* = 0.00025

Abbreviation: UK, United Kingdom.

### Ketamine use

Participants reported first trying ketamine at an average age of approximately 19 years, with a range from 12 to 50 years across all participants (Table 1). The most common route of administration was snorting (93%), followed by injecting (2%) and ‘boofing’ (administration per rectum, 1%). On average, participants consumed 2.0 g of ketamine per day, with treatment‐seeking participants reporting higher average use (M = 2.67 g, where M denotes mean) than non‐treatment‐seeking users (M = 1.68 g), ranging up to 20 g at a time being the highest daily dose reported. The initial source of ketamine was typically friends (72%), followed by dealers (14%). Four participants reported first obtaining ketamine through a clinical prescription. For most participants (73%), ketamine was their primary drug of use. The length of time participants reported problematic use ranged from 2 months to 10 years.

Among both treatment‐seeking and non‐treatment‐seeking participants, the primary initial motivations for using ketamine were dissociation (73%), self‐medication (53%) and the appeal of its psychedelic effects (56%). At the time of the survey, the primary reasons for continued use were dissociation (57%), self‐medication (50%) and relaxation (40%).

Regarding their reasons for initial use, one participant said:

‘I began in a clinical setting and have only acquired K via prescription from a mental health practitioner…I now have a telehealth provider who prescribes me nasal spray. Instead of using the spray as intended, I take appropriate sterilization steps to do at home IM [intramuscular] injections.’

### Symptoms of problematic ketamine use

When asked about physical symptoms related to ketamine use, 13% reported no symptoms. However, a significant number experienced bladder problems (60%), nasal issues (60%), ‘K‐cramps’ (56%), headaches (17%) and other symptoms (12%). Within the ‘other’ category, specific symptoms included kidney issues, gall bladder issues, body aches, pancreas issues and liver issues, among others such as heart palpitations, erectile dysfunction, blood in urine, constipation and brain fog.

In response to these symptoms, the majority (56%) did not seek treatment. Of those who did, 26% went to accident and emergency, 25% consulted their doctor, 12% saw an urologist and 6% sought other types of care, including self‐management and gallbladder removal. Satisfaction with treatment of these physical symptoms was low, with only 36% reporting feeling satisfied. When asked for further elaboration of treatment experiences, qualitative analyses revealed several cited a lack of effective treatment options or only receiving pain relief (Table 2).

**TABLE 2:** Brief thematic analysis for the treatment experiences of physical symptoms of KUD (*n* = 46).

Theme	Example quote	Frequency (*n*)	Percent (%)
Discharged without treatment	‘I attended A&E with my bladder feeling like it was about to burst but no urine was coming out for 5 and a half hours I was stuck in crippling pain on the toilet with sharp stabbing pains in my vagina anytime that I tried to push, I attended A&E and was sent home’	10	22
Only offered was pain relief	‘All they did was give me painkillers and send me on my way’	8	17
Health care professionals did not know what to do	‘I was prescribed an antihistamine by the bladder doctor, and it was not explained why. When I followed up the staff were unclear what the reason for the prescription was.’	7	15
Left on a waiting list	‘Still waiting for a GP appointment’	7	15
Doctors did not understand	‘GP does not think ketamine is addictive, just told me to stop, does not have a clue’	5	11
Judgement	‘I was never honest about why I was experiencing bladder problems because I was too embarrassed to say I have been abusing drugs’	5	11
Good experience	‘A nurse recommended a local addiction service. Throughout my time there I accessed therapy for my BPD, one of the root causes of the issue, and learned everybody's journey to sobriety is not the same, and it was ok to slip up’	4	8

Abbreviations: A&E, accident and emergency; BPD, bipolar disorder; GP, general practitioner; KUD, ketamine use disorder.

### Abstinence syndrome/withdrawal symptoms

During periods of abstinence, the most commonly reported withdrawal symptoms were cravings (71%), low mood (62%), anxiety (59%) and irritability (45%). Other frequently reported symptoms included sleep disturbances (36%), fatigue (34%), abdominal pain (34%) and insomnia (32%). Less common symptoms included poor appetite (24%), sweating (23%), shaking (17%), palpitations (12%), dysphoria (12%), tremors (9%) and delusions (9%). Additional symptoms reported by some participants included mania, bladder pain and negative psychological impacts.

### KUD treatment

Among our survey population, 41% had sought treatment for KUD. Among those who sought addiction treatment, 38% attempted to get treatment once, 27% twice, 10% three times, 9% four times and 16% more than five times.

Regarding where they accessed treatment, 56% went to an addiction treatment provider, 36% consulted their doctor, 30% used an addiction charity and 28% accessed treatment through other sources, including private therapy and university services.

The most common treatment methods participants had undergone included outpatient therapy (58%), support groups (57%), inpatient rehabilitation (28%) and holistic therapies (16%). Additionally, 17% chose other treatment methods, with responses including exercise and harm reduction techniques. The most effective treatment was reported to be support groups (22%), followed by inpatient rehabilitation (20%), outpatient therapy (15%) and holistic therapies (4%). However, the most frequent response was ‘other’ (38%), with people most commonly responding that no treatment was effective (13.5%). Other responses included developing hobbies, talking to others with KUD and quitting alone.

### Facilitators to treatment

Among treatment‐seeking users, participants cited health problems related to ketamine use (66%) and being ‘sick of the lifestyle’ (64%) as the primary reasons for seeking treatment. Other reasons included pressure from friends/family (48%), financial concerns (38%), a desire to reduce use (38%), seeking abstinence (37%), wanting ‘time out’ (28%) and legal reasons (9%). Additionally, 20% cited other reasons, with *n* = 9 reporting that ketamine had a very negative impact on their life, for example, ‘I lost all of my close friends, my partner, my house, had 2000k [sic] debt, dropped out of college four times‐ it ruined my life and just had enough.’

When asked about the most important factors when choosing a treatment program, cost/affordability was the most cited factor for both treatment‐seeking (73%) and non‐treatment‐seeking (72%) participants. For treatment‐seeking participants, other important factors included the treatment approach used (73%), location/ease of access (67%) and privacy/confidentiality (60%). Non‐treatment‐seeking participants also emphasised privacy/confidentiality (60%) and treatment approach used (54%).

Treatment‐seeking users said the most important factor that helped them access treatment was family/friend support (59%), availability of treatment (41%) and education/awareness of treatment options (23%). A total of 23% chose other, including no factors helped, proximity to home, peer support within treatment, desperation, university support, confidentiality reassured and health scare. Respondents were also asked what would be important to include in a KUD treatment service, with many highlighting the need to educate healthcare professionals (Table 3).

**TABLE 3:** Brief thematic analysis of suggestions from treatment‐seeking users for methods to improve treatment services for KUD (*n* = 77).

Theme	Example quote	Frequency (*n*)	Percent (%)
Educating care workers about ketamine	‘Education of health professionals including GPs, A&E staff, Police, Social workers.’	20	26
Addressing the physical issues	‘I think they need to research precisely into drugs and other options which would help fight K bladder, K kidneys and K stomach cramps because ketamine can make your lifespan much shorter.’	10	13
Address mental health issues	‘Focus on the mental health side of things as well as the addiction.’	9	11
Surrounded by other ketamine users/ex‐users	‘Perhaps similar stories of people so you do not feel so alone. Stories of people who have beaten it and gotten better.’	6	7
Easy to access	‘Push in GP surgeries highlighting how easy taking the first steps are.’	6	7
Harm reduction/damage control	‘Reduction and harm minimizing advice at the front end.’	4	5
Affordable	‘Free services.’	3	3
Counselling	‘Actually understanding why I use would help.’	2	2
Ketamine specific	‘A tailored service, staff have very high awareness of ketamine, including on physical health etc.’	2	2
Option to ease out of ketamine use	‘Alongside gradual use reducing.’	2	2
Outpatient options	‘Have options that accommodate the lifestyle of the user, if they need to work offer Intensive Outpatient.’	2	2
Removing the stigma	‘Getting rid of the stigma around ketamine. People only think you deserve help for drugs when it's heroin.’	2	2
Drug testing	‘Testing and checking.’	2	2

Abbreviations: A&E, accident and emergency; GP, general practitioner; K, ketamine; KUD, ketamine use disorder.

Non‐treatment‐seeking users answered what specific approaches they would want in a KUD treatment, which revealed an emphasis on therapy and using medication to quit ketamine (Table 4).

**TABLE 4:** Brief thematic analysis of suggestions from non‐treatment‐seeking users for improving access to treatment (*n* = 38).

Theme	Example quote	Frequency (*n*)	Percent (%)
Therapy	‘Talk therapy to find route cause of addiction.’	6	16
Medicated assistance	‘Infusions.’	6	16
Harm reduction	‘Focus on symptom reduction rather than outright “detoxing” that might be used for stimulants or depressant drugs.’	5	13
Holistic approaches	‘Holistic approaches yoga meditation.’	3	8
Education	‘More education on the dangers of use.’	3	8
Psychedelic assisted therapy	‘Psychedelic assisted therapy, Maybe even using ketamine.’	3	8
Manage physical symptoms	‘A lot of people who end up relapsing end up doing so due to needing to manage physical symptoms (especially K‐cramps) rather than due to a particular “want” to do so.’	3	8
More awareness	‘More awareness of treatment methods’	3	8
Less stigma	‘Less stigma.’	2	5
Community support	‘Community‐based management would be preferable.’	2	5

### Barriers to treatment

The most common barrier preventing non‐treatment‐seeking users from seeking treatment was the desire to continue using ketamine (60%).

This was followed by: fear of judgement/stigma (42%), concern of being found out by family/friends/employer (35%), lack of motivation to quit/happy with lifestyle (33%), lack of awareness of available options (29%), financial constraints (28%), concerns about privacy/confidentiality (25%), and believing treatment will not be effective (25%).

### Perceptions of KUD awareness

Treatment‐seeking participants reported that the services they used had little (31%) or some (31%) awareness of ketamine, were not tailored to ketamine use (43%) and were generally only somewhat effective (43%). Table 5 shows a qualitative analysis of respondents' perceptions of KUD services, including a lack of understanding within healthcare and treatment being too broad.

**TABLE 5:** Brief thematic analysis of the awareness of KUD among treatment services (*n* = 21).

Theme	Example quote	Frequency (*n*)	Percent (%)
Healthcare providers do not understand ketamine	‘They do not understand.’	8	38
Treatment is not specific to ketamine	‘The chances of rehab being tailored to what I get from ketamine is slim. The ketamine experience involves frequent near‐death experiences and out of body hallucinations. Ketamine provides a very spiritual experience. This is something I have struggled to let go of for years. No treatment services have ever mentioned this aspect.’	8	38
People do not think ketamine is addictive	‘It is hugely underestimated, being a psychological addiction on paper, people assume it's a minor addiction and as easy as giving up cannabis. I would argue both from personal experience and a plethora of anecdotes from other long‐term abusers, that the physical symptoms that occur when abstaining after a long period of heavy use can be comparable to physical withdrawal symptoms from substances such as heroin, alcohol or benzos.’	4	19

Abbreviation: KUD, ketamine use disorder.

All participants were asked if there was sufficient awareness in education and peer groups about the risks associated with ketamine use (*n* = 195). The majority (59%) responded ‘definitely not’, with 27% saying ‘probably not’, 9% unsure and only 3% responding ‘probably yes’. Qualitative analysis (Table 6) revealed a common theme of insufficient information and awareness regarding the physical effects of ketamine use.

**TABLE 6:** Brief thematic analysis about awareness of risks associated with ketamine use (*n* = 195).

Theme	Example quote	Frequency (*n*)	Percent (%)
Lack of information	‘No one even understands what ketamine is or what it does. It should not be our job to explain the science of ketamine, it should be taught, and people need to be educated on it as it gets so much less information, as cocaine for example.’	80	41
Lack of awareness of physical effects	‘People need to be informed about the problems it can cause with your bladder, kidney, if you get to daily usage of grams or more.’	28	14
Lack of awareness of addictive potential	‘People know the risks about Heroin and Cocaine, but not how strong the addiction to Ketamine can become even stronger than being addicted to Heroin or Cocaine.’	28	14
Lack of awareness in professionals	‘Not taken seriously as just seen as a party drug.’	17	8
Ketamine can affect your mental state	‘I pointed out that K is not a harmless drug and opened up about my struggles over the years and recent struggles which had me suicidal over a few months.’	16	8
New generation of drugs	‘I feel it is the heroin of a generation, and more information will become available once more time passes and more people my age begin to suffer so greatly from misuse that it cannot be hidden anymore.’	10	5
Lack of awareness (general)	‘There is little support for getting a health assessment as GPs believe this should fall on adult services.’	9	4
Clinical advancements of ketamine contribute to addiction	‘Ketamine therapy and depression globalisation is eliminating awareness, danger of addiction. It's a gateway drug which will not truly solve any problem among mental health.’ ‘In the US, it's currently being pushed as a miracle drug. Its addictive potential is often overlooked and there's very little research on it.’	6	3

Abbreviations: GPs, general practitioners; K, ketamine; US, United States.

One user reports: ‘Ketamine therapy and depression globalization is eliminating awareness, danger of addiction. It's a gateway drug which truly won't solve any problem among mental health.’

## DISCUSSION

As ketamine use continues to increase both clinically and recreationally, developing effective treatments for KUD has become increasingly urgent. This study reveals several under‐reported symptoms associated with problematic ketamine use, observed in both treatment‐seeking and non‐treatment‐seeking individuals. Moreover, the findings expose significant gaps in our current understanding of KUD and highlight the shortage of effective treatment strategies, offering patient‐centred recommendations to improve accessibility and treatment outcomes.

### Symptoms of KUD and abstinence syndrome

Our survey revealed that a significant proportion of users experience physical symptoms linked to problematic ketamine use. While previous research reported that approximately one‐third of frequent users suffer from ‘K‐cramps’ [16], our data show that 60% of participants report these symptoms, nearly double the previous estimate. This discrepancy may arise from differences in data collection methods, with our anonymous survey likely encouraging more honest reporting compared to previous face‐to‐face interviews.

In addition to ‘K‐cramps’, our study confirms the presence of an abstinence syndrome associated with ketamine cessation. Earlier studies identified withdrawal symptoms such as fatigue, anxiety, dysphoria, cravings and palpitations [17, 18], which resemble those reported during opiate withdrawal [19]. Our findings corroborate these symptoms and reveal additional ones, including sleep disturbances, shaking and delusions. The identification of these new symptoms may be attributed to the larger sample size used in this study, which has helped capture new insight into abstinence syndrome and highlight the inconclusive understanding of ketamine cessation in KUD.

It is important to note that participants selected withdrawal symptoms from a predefined list, including insomnia and sleep disturbances, without further clarification of the differences between these two items. This lack of distinction may have resulted in overlapping responses. Moreover, despite its clinical uses for the treatment of depression, KUD is associated with psychological issues such as depression, psychosis and cognitive impairment, which persist independently of withdrawal in some [20, 21, 22], but not all [12] studies. Therefore, a future comprehensive understanding of ketamine's impact on users should include these broader psychological effects.

### Attitudes to treatment

Our study explored treatment preferences among both treatment‐seeking and non‐treatment‐seeking individuals. Aside from therapy, respondents expressed a desire for harm reduction strategies and medication‐assisted interventions to facilitate gradual reduction in ketamine use. A recent review of pharmacological approaches to treating KUD has highlighted a lack of extensive research, but noted potential benefits from interventions such as benzodiazepines for withdrawal management and naltrexone for craving and relapse prevention [23]. However, the majority of existing evidence comes from case studies, emphasising the need for robust clinical trials to establish the efficacy of these treatments.

A significant finding from our study is the widespread lack of awareness about KUD among medical professionals and the general public. Many participants noted that the growing emphasis on ketamine as a therapeutic agent may have obscured its abuse potential. Indeed, a small but noteworthy number of participants in this study first accessed ketamine through a clinical prescription in the US, reinforcing concerns that its clinical uses may downplay awareness of the risks.

This study reveals there is a pressing need for increased awareness and education about KUD. Such education could help deter use, encourage harm reduction practices and enhance the ability of professionals to effectively treat and advise individuals with problematic use. One participant noted that, unlike other drugs of abuse such as heroin and cocaine, ketamine is comparatively overlooked in terms of its addictive potential. Another user even describes ketamine as the ‘heroin of our generation,’ indicating that the older generations may not fully grasp the risks associated with the drug, particularly as the average ketamine user is considerably younger than those who use substances like heroin [24].

Moreover, this study highlights the need for accessible and affordable treatment options. More local community support groups and increased outreach and education from treatment providers (charity, non‐profit or National Health Services) about available free or low‐cost options are crucial. Given the younger demographic of ketamine users, targeting through social media may be effective. Our findings show that affordability is a key factor in treatment choice, and services that integrate easily into users' daily lives, such as those that allow continued employment, could improve engagement and adherence to treatment. Additionally, support groups specifically addressing ketamine‐related issues, such as the ‘K‐hole’ and out‐of‐body experiences, could offer significant benefits. Future research should focus on designing targeted group therapy for KUD, considering the unique experiences and challenges faced by ketamine users, and examine the attitudes of treatment providers to inform service development.

### Limitations and future directions

This study provides a comprehensive exploration of the experiences of individuals with problematic ketamine use, with the aim of informing more effective treatment approaches. Nonetheless, several limitations must be acknowledged. The primary recruitment method, via Reddit subreddits dedicated to ketamine addiction, may have introduced selection bias as participants from these platforms might have more severe negative experiences than the broader ketamine‐using population. Additionally, there was a high attrition rate, likely because of the length of the questionnaire and the absence of incentives. This may have influenced data quality and introduced bias, as those who completed the survey could have been the most affected or frustrated by KUD. Consequently, this may have resulted in the under‐representation of individuals with less severe or more positive experiences, including those who may be prescribed ketamine and attribute their recovery to its use, despite potential prescription misuse. Additionally, we analysed all available responses, including participants with missing data, maximising data retention, but potentially introducing bias because of varying sample sizes and differential question skipping. The use of a Likert scale for treatment effectiveness may have also resulted in varied interpretations. Future studies should explore treatment effectiveness in different domains, including symptom reduction, improved quality of life, sustained abstinence, as well as outcome differences across demographics such as age, gender and comorbid psychiatric conditions.

To address these limitations, subsequent research should use diverse recruitment strategies, such as outreach at festivals and engagement with medical professionals who prescribe ketamine. Additionally, polydrug use is another critical factor to consider, as over a quarter of our participants reported using substances other than ketamine as their primary drug. Research indicates that polydrug use is linked to increased mental distress and poorer treatment outcomes [25]. Given that ketamine‐related fatalities frequently occur in polydrug contexts, understanding its interactions with other substances is vital for developing comprehensive treatments [26]. Prospective studies should collect detailed data on substance use, including frequency, dosage and combinations, to better characterise polydrug use in KUD. Where possible, studies should compare ketamine users with groups that exhibit similar polydrug use, but without ketamine. Furthermore, research should explore symptoms in extreme cases involving high doses or unusual routes of administration.

## CONCLUSION

This study highlights the significant physical and psychological risks associated with problematic ketamine use and identifies barriers to effective treatment. Our findings underscore the need for improved treatment programs and increased awareness among healthcare professionals and the public. By focusing on evidence‐based treatments, such as specialised group therapy and pharmacological interventions, we can enhance treatment outcomes and address the growing issue of KUD. Moreover, targeted educational initiatives for both the general public and treatment providers, alongside harm reduction policies, could further help improve outcomes for those with KUD. Future research should continue to explore these areas to refine treatment approaches and better support individuals affected.

## AUTHOR CONTRIBUTIONS

**Rebecca E. Harding:** Conceptualization (lead); methodology (lead); writing—original draft (lead). **Tamsin Barton:** Data curation (equal); formal analysis (equal). **Maeve Niepceron:** Data curation (equal); formal analysis (equal). **Ella Harris:** Data curation (equal); formal analysis (equal). **Emily Bennett:** Data curation (equal); formal analysis (equal). **Emily Gent:** Investigation (equal); project administration (equal). **Flora Fraser:** Investigation (equal); project administration (equal). **Celia J. A. Morgan:** Conceptualization (lead); funding acquisition (lead); supervision (lead); writing—review and editing (lead).

## DECLARATIONS OF INTERESTS

None.

## Supporting information


**Data S1.** Supplementary Information.

## Data Availability

The data that support the findings of this study are available on request from the corresponding author. The data are not publicly available due to privacy or ethical restrictions.
