# Supplementary material for: The landscape of ketamine use disorder: Patient experiences and perspectives on current treatment options
Source: Addiction. 2025 Apr 28;120(10):1970–9. doi: 10.1111/add.70073 (PMC12426348; doi:10.1111/add.70073)
Supplement: Supplementary file 1 — Data S1. Supplementary Information. [file ADD-120-1970-s001.docx]

# Supplementary

**S1. Survey Questions**

1. What is the date today?
2. Do you consider yourself to have a problem with your ketamine use? (Selected choice - No, Current, Previous)

***Demographics (12 questions)***

1. Where did you hear about the survey? - Selected Choice/Other (Text)
2. How old are you?
3. What country do you live in? - Selected choice/ Other (Text)
4. What country/borough do you live in?
5. Which of the following most accurately describes you? (gender) - Selected choice/Text
6. Have you been diagnosed with a mental health disorder? (If yes, please specify) - Selected choice/ Other (Text)
7. How frequently do you use ketamine? Please indicate in numbers (0-7) the number of days per week you use (or used if currently abstinent) ketamine?
8. On a day that you use ketamine, how much would you use on average in grams? (Use 0.5 if a half for example, 1 for one gram)
9. How do you take ketamine? - Selected choice/ Other (Text)
10. How many days ago did you last take ketamine? (Please answer with a number. For example: 0 = today, 3 = three days ago; 180 days = 6 months ago, etc.)
11. For how long has your use of ketamine been problematic? Or if you no longer have a problem with ketamine use, for how long was your ketamine use problematic? (Please answer with a number. For example: 6 months, 1 year, 7 years)
12. Is ketamine the primary drug that you use? If no, please conclude which other drugs you use in your text box - Selected choice/ Other (Text)

***SDS (ex-users) (5 questions)***

1. Thinking back to when you had a problem with ketamine use, do you think your use of ketamine was out of control? - Selected choice
2. Would the prospect of missing a fix (or dose) make you anxious or worried? - Selected choice
3. Did you worry about your use of ketamine?- Selected choice
4. Did you wish you could stop? - Selected choice
5. How difficult did you find it to stop or go without ketamine? - Selected choice

***SDS (current users) (5 questions)***

1. Do you think your use of ketamine is out of control? - Selected choice
2. Does the prospect of missing a fix (or dose) make you anxious or worried? - Selected choice
3. Do you worry about your use of ketamine?- Selected choice
4. Do you wish you could stop? - Selected choice
5. How difficult would you find it to stop or go without ketamine? - Selected choice

***Attitudes towards ketamine and addiction (11 questions)***

1. Do you think there is currently enough awareness around issues with ketamine use? i.e., through education in school or amongst your peer group - Selected choice
2. Please use the text box to further explain - text
3. What did you initially find appealing about ketamine as a drug? Please select all that apply - Selected choice/ Other (text)
4. How old were you when you first tried ketamine? - text
5. How did you first source ketamine? - Selected choice/ Other (text)
6. What are the main reasons for your use of ketamine now? Please select all that apply - Selected choice/ Other (text)
7. When you stopped using ketamine or went without it for a long period, did you experience any of the following? Please select as many that apply - Selected choice/ Other (text)
8. Do you experience any physical symptoms as a result of your use of ketamine? Please select all that apply - Selected choice/ Other (text)
9. Did you seek any treatment for the physical symptoms associated with ketamine consumption? If yes, from where? - Selected choice/ Other (text)
10. Were you satisfied with the treatment you received?- Selected choice
11. Please use the text box to further explain –text

***Treatment-seeking (1 question)***

1. Have you ever sought treatment for ketamine use?

***Yes – treatment-seeking (4 questions) - Treatment-seeking only***

1. How many times in the past have you sought treatment to reduce your ketamine use? -Selected choice
2. Are you currently seeking treatment? If so, for how long? - Selected choice/ Other (text)
3. What type of treatments have you tried? Please select all that apply - Selected choice/ Other (text)
4. Which treatment was the most effective? - Selected choice / Other (text)

***Attitudes towards ketamine services (12 questions) – Treatment-seeking only***

1. What were your primary reasons for seeking treatment? Please select all that apply - Selected choice/ Other (text)
2. Where did you go to seek treatment? - Selected choice/ Other (text)
3. Did you feel like treatment services are aware of ketamine addiction? – Selected choice
4. Do you feel like the treatment you were offered was adequately tailored to ketamine addiction? – Selected choice
5. How effective do you feel the treatment(s) you received were in addressing your ketamine addiction? – Selected choice
6. Please use the sliding scales to represent how much you agree with the following statements about ketamine addiction treatment options available to you:
   a. The treatment options are effective
   b. The treatment options are accessible
   c. The treatment options are affordable
   d. There is stigma around the use of ketamine
   e. There is lots of available information about treatment options
   f. I trust the treatment providers
7. Use this box to discuss any other perceptions of ketamine services – text
8. What factors would you consider important when choosing a treatment program for ketamine addiction? Please select all that apply - Selected choice/ Other (text)
9. What challenges or barriers did you face while accessing ketamine addiction treatment? (Select all that apply) - Selected choice/ Text
10. What factors helped you access treatment? Please select all that apply - Selected choice/ Text
11. What do you think would be important to include in a treatment service for ketamine users? - Text
12. Is there anything else you would like to share about your experiences or thoughts on ketamine addiction treatment? - Text

**Attitudes of non-treatment seeking users (8 questions) - Non-treatment seeking only**

1. What factors, if any, have deterred you from seeking ketamine addiction treatment? Please select all that apply – Selected text/ Other Text
2. What factors would you consider important when choosing a treatment program for ketamine addiction? Please select all that apply - Selected choice/ Other (Text)
3. Do you feel like treatment services are aware of ketamine addiction? - Selected choice
4. Please use the sliding scales to represent how much you agree with the following statements about ketamine addiction treatment options available to you:
   a. The treatment options are effective
   b. Treatment options are accessible
   c. The treatment options are affordable
   d. There is stigma around the use of ketamine
   e. There is lots of available information about treatment options
   f. I trust the treatment providers
5. Use this box to discuss any other perceptions of ketamine treatment services – text
6. Are there any specific treatment approaches or methods you would prefer for ketamine addiction treatment? - text
7. What additional support or services would you like to see offered as part of ketamine addiction treatment programs? - text
8. Is there anything else you would like to share about your experiences or thoughts on ketamine addiction - text

**Results**

*S2. Participant demographics for all survey responders*

| **Characteristics (N=274)** | **%** |
| --- | --- |
| **Gender** |  |
| Male | 47.67% |
| Female | 44.19% |
| Trans-male | 4.26% |
| Non-binary | 3.10% |
| Other | 0.78% |
| **Mental health diagnosis** | 58.87% |
| Depression | 58.97% |
| Anxiety | 45.51% |
| BPD/EUPD | 16.03% |
| C/PTSD | 12.82% |
| Bipolar | 7.69% |
| Eating disorder | 5.77% |
| OCD | 5.77% |
| Gender dysphoria | 2.56% |
| Psychosis | 1.92% |
| Schizophrenia | 1.92% |
| Panic disorder | 1.28% |
| **Location** |  |
| United Kingdom | 75.28% |
| United States | 11.54% |
| Europe | 3.08% |
| Canada | 1.15% |
| Australia | 0.77% |
| Other/unspecified | 1.15% |
